# Lipid composition and mechanical force underlie multi-modal regulation of Piezo1 gating

**DOI:** 10.1126/sciadv.aed7115

**Published:** 2026-06-03

**Authors:** George Vaisey, Roderick MacKinnon

**Affiliations:** Laboratory of Molecular Neurobiology and Biophysics, Howard Hughes Medical Institute, The Rockefeller University, New York, NY 10065, USA.

## Abstract

Piezo1 ion channels are widely expressed cellular mechanosensors. They adopt an intrinsically curved shape when closed and are thought to open when mechanical forces applied to the membrane favor a more flattened conformation. In previous studies, Piezo1 channels in lipid vesicles adopted a somewhat flattened conformation mediated by membrane curvature; however, the ion conduction pore remained closed. In line with the closed pore, Piezo1 channels do not open and conduct ions in the kind of lipids that were used in the structural studies. Here, we show first that Piezo1 channels in cell-derived membranes retain the ability to open and conduct ions under mechanical force, and second, that in cell-derived membrane vesicles, they adopt a more completely flattened disk shape associated with large conformational changes within and around the ion conduction pathway. These conformational changes occurring in cell-derived lipid membranes suggest that mechanical force is necessary but insufficient, and that a specific membrane-derived cofactor complements mechanical force to activate Piezo1.

## Teaser

Piezo1 under mechanical force adopts a unique conformation that depends on lipid membrane composition.

## INTRODUCTION

Many living cells depend on rapid responses to mechanical cues. Piezo ion channels are on the frontline of this sensory process in mammalian cells (*1*–*4*). Structural studies of Piezo1 in detergent micelles or in liposomes show that it forms a trimeric assembly that is intrinsically curved under resting conditions, but that it can be flattened under applied force (*5*–*9*). If flattening were coupled to pore opening, as the current model of Piezo1 mechanical gating posits, then we would have a simple description of mechanical gating, whereby lateral membrane tension would open the pore by favoring the flattened, in-plane expanded conformation. Unfortunately, an important piece of this explanation is missing, because so far the flattened structure has a closed pore. Perhaps related to this missing piece is the finding that Piezo1 fails to activate, i.e., open functionally, in membranes comprising the kind of lipids used in structural studies.

Compared to other mechanosensitive channels, including the prokaryotic MSCs or eukaryotic two-pore potassium channels (K2Ps), Piezo channels have in general remained refractory to robust functional studies in reconstituted systems (*10*–*14*). Spontaneous channel activity attributed to Piezo1 has been reported in lipid droplet bilayers and membrane patches of proteoliposomes (*15*, *16*), but reversible mechanically activated currents have not been demonstrated. Moreover, the measured single-channel conductance in lipid droplet bilayers is approximately four times higher than patch-clamp recordings of endogenous and overexpressed Piezo1 in the presence of millimolar concentrations of calcium (*1*, *17*–*21*) and approximately twice the value of recordings made in the absence of calcium (*22*, *23*). Here, we show first that Piezo1 ion channel activity is maintained in vesicles released from *N*-ethylmaleimide (NEM) treatment of cells. Then we determine structures of Piezo1 channels in vesicles derived from these same membranes under varying membrane bending forces applied through the curved vesicle membrane. The structures exhibit a unique flattened conformation associated with changes that map directly to channel activation and imply a multi-modal basis for Piezo1 channel gating.

## RESULTS

### Cell membrane–derived vesicles retain Piezo1 channel function

**Fig. 1.**
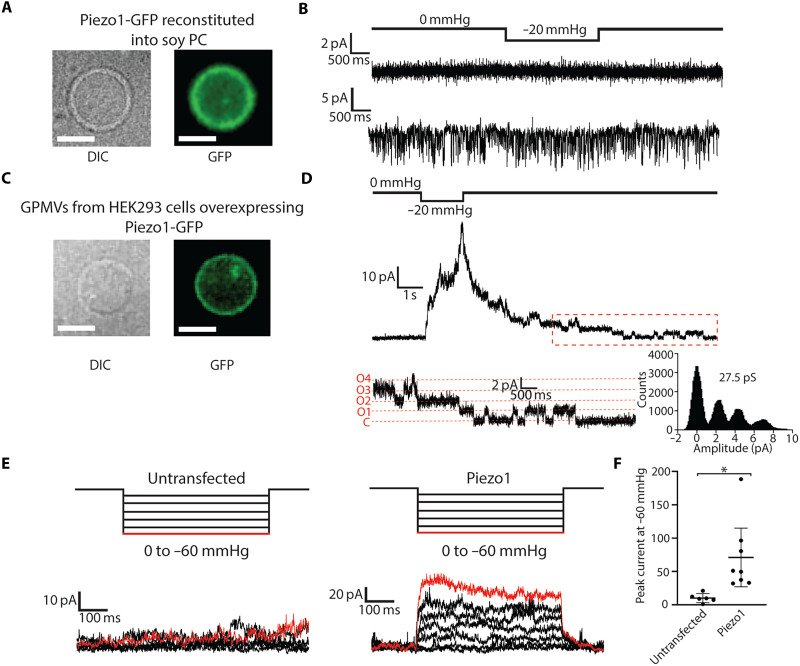
Functional mechanical activation of Piezo1 in GPMVs but not when reconstituted into soy PC liposomes. (**A**) Differential interference contrast (DIC) (left) and fluorescence image (right) of purified Piezo1-GFP reconstituted into soy PC liposomes that were then swelled into GUVs. Scale bars, 10 μm. (**B**) Two example patch-clamp recordings of patches excised from GUVs as prepared in (A). Recordings are made holding at −80 mV with a 2-s pulse of −20 mmHg. (**C**) Images taken as in (A) of GPMVs generated by NEM incubation of cells overexpressing Piezo1-GFP. (**D**) Electrical recording of a patch excised from a GPMV overexpressing Piezo1-GFP. The patch is held at +80 mV, and a 1.5-s pressure pulse at −20 mmHg is made to elicit Piezo1 channel openings. The red-dashed inset shows a 5-s duration of single-channel openings, and the corresponding amplitude histogram is shown on the right, giving a single-channel conductance of 27.5 pS. (**E**) Excised patch recordings of GPMVs from Piezo1 knockout HEK293 cells that were either untransfected or overexpressing Piezo1. Patches were excised and held at +60 mV with increasing −10-mmHg pressure steps up to −60 mmHg. (**F**) Data from recordings as in (E) plotted for untransfected (*n* = 6) or Piezo1-overxpressing (*n* = 8) GPMVs, *P = 0.0135, as assessed by an unpaired *t* test with Welch’s correction.

We first reconstituted Piezo1 channels into small unilamellar vesicles (SUVs) consisting of defined lipid compositions used in past structural analyses of Piezo1 in membranes (fig. S1) (*8*). From the SUVs we generated giant unilamellar vesicles (GUVs), microns in size, using established protocols (*24*, *25*) and confirmed incorporation of Piezo1 by detecting the fluorescence of a C-terminally fused green fluorescent protein (GFP) (Fig. 1A). Although Piezo1 channels were present in the membrane, we could not detect channel openings by patch recording (Fig. 1B). We varied Piezo1 purification conditions, lipid composition, and conditions for forming GUVs, all to no avail regarding channel activity (table S1). We occasionally observed transient ionic currents of varying amplitudes that were not reproducibly mechanically activated by patch pressurization (Fig. 1B). As we have found in other studies, the reconstitution of membrane proteins at high concentrations can yield this kind of channel-like activity (*26*), but without the correct single-channel conductance and mechanical activation, these do not represent bona fide ion channel recordings.

Exploring alternative approaches, we experimented with the formation of plasma membrane vesicles (PMVs) by incubation of cells with NEM in the presence of millimolar free calcium (*27*, *28*). Adherent human embryonic kidney (HEK)–293 cells treated with NEM generate giant PMVs (GPMVs), as shown in Fig. 1C for cells overexpressing mPiezo1-GFP. Electrical recordings of membrane patches excised from GPMVs yielded reversibly pressure-activated currents. When channels were few enough to detect single-channel events, we measured the single-channel conductance to be about 27.5 pS (Fig. 1D), consistent with cellular recordings of Piezo1 by us and others (*1*, *17*–*21*). Previous studies of purified Piezo1 reported single-channel conductance values of 110 to 130 pS, which were attributed to the experimental conditions, including the absence of divalent cations and high salt concentrations (*15*). In inside-out patches excised from cells overexpressing Piezo1, we find that the single-channel conductance approaches a maximum value at ~30 to 40 pS in physiological potassium chloride conditions (fig. S2), similar to measurements made by others (*22*). Recordings of Piezo1 from GPMVs display near-identical single-channel conductance values to measurements made from excised cell patches (fig. S2).

Macroscopic recordings of GPMVs derived from cells overexpressing Piezo1 showed increases in current in response to stepwise pressure pulses, with a mean peak current amplitude at −60 mmHg of 71 ± 49 pA recorded at +60 mV (Fig. 1, E and F). By contrast, we did not observe substantial pressure-activated currents in GPMVs from Piezo1 knockout cells (Fig. 1, E and F). There are some notable differences in Piezo1 function when electrically recorded in GPMVs compared to intact cell membranes. Piezo1 currents in GPMVs do not inactivate like in cells (fig. S3). This is not due to NEM modification of free cysteines in Piezo1 as channel inactivation is maintained in cell-attached patches after 1 hour treatment of cells with NEM (fig. S3). In addition, while Piezo1 can be robustly mechanically activated in GPMVs, we do not observe maximal activation of currents at −80-mmHg pressure, in contrast to recordings in cell-attached patches. Together, the electrical recordings show that mechanical activation of Piezo1 is maintained in GPMVs, but with a change in the pipette pressure at which maximum activation occurs. These differences could be due to changes in membrane composition or the loss of membrane-bound cytoskeleton upon GPMV formation, both of which have been shown to influence Piezo1 channel activity by others (*23*, *29*–*31*). We next examined whether Piezo1 structures in cell-derived membranes, which support function, appear different than Piezo1 structures in reconstituted membranes, which do not support function.

### Determination of outside-in and outside-out Piezo1 structures in cell-derived membranes

**Fig. 2.**
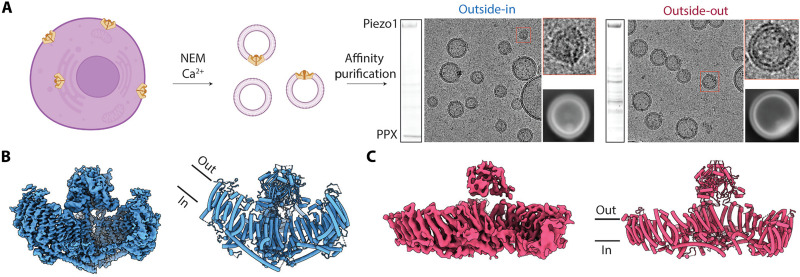
Methodology to isolate outside-in and outside-out Piezo1 in PMVs and determine single particle cryo-EM structures. (**A**) Left: Schematic illustrating basic approach of obtaining Piezo1-containing PMVs. Right: Coomassie-stained denaturing gels, with annotations for the Piezo1 and PreScission protease (PPX) band and representative micrograph images of the final outside-in and outside-out Piezo1 PMV samples, along with a representative 2D class image. (**B**) Cryo-EM map (left) and structure of outside-in Piezo1 in PMVs. (**C**) Cryo-EM map (left) and structure of outside-out Piezo1 in PMVs.

To determine Piezo1 structures in vesicles derived from the PMVs, we used an approach developed in our laboratory to study other ion channel structures (*32*), but with modifications to overcome lower levels of Piezo1 expression and to selectively bias preparations toward outside-out and outside-in orientations of the Piezo1 channel (Materials and Methods). In brief, after NEM treatment of suspension HEK293 cells, centrifugation was used to separate the GPMVs from what we call small PMVs (SPMVs), whose size is reduced further by gentle sonication for cryo–electron microscopy (cryo-EM). After observing that the SPMVs contained Piezo1 in both outside-in and outside-out orientations in electron micrographs, we used affinity purification methods targeting either an intracellular GFP tag or an extracellular ALFA tag to separately enrich the two populations of channels for single-particle cryo-EM data collection and processing. In both cases, the final preparation of SPMVs showed a dominant Piezo1 band on SDS–polyacrylamide gel electrophoresis after Coomassie staining (Fig. 2A). SPMVs with Piezo1 adopting the outside-in orientation exhibited a tear-drop shape owing to membrane distortion created by the curved Piezo1 channel, as was reported previously (Fig. 2A) (*6*, *8*, *33*, *34*). Processing of the outside-in dataset using SPMVs in the size range 11- to 20-nm vesicle radius, yielded a structure at 3.7-Å resolution (figs. S4 and S5). This resembled the curved structures of Piezo1 previously determined in detergent micelles and in liposomes (*5*, *6*, *8*), with a mean radius of curvature of about 11.8 nm (Fig. 2B and fig. S5). The refined structural model contains 1352 residues (of 2547), with the first four-transmembrane repeats and flexible loops missing. We note that this structure, same as outside-in structures in small vesicles determined before, is highly curved.

Outside-out Piezo1 SPMVs by contrast exhibit a different shape on micrographs, where the opposing curvature of the vesicle relative to the intrinsic curvature of Piezo1 produces a bending force that completely flattens the channel into a conformation in which the transmembrane arms are coplanar to the bilayer (Fig. 2, A and C, and figs. S6 and S7). Despite similar sample quality and data acquisition settings to our outside-in map, the outside-out map reaches only a resolution of ~6 Å (fig. S5). Even at this lower resolution, the map defined a region of the protein with sufficient detail to build a Cα model extending from residues 589 to 2547 (Fig. 2C).

### Conformational differences between curved and flat Piezo1 channels in cell-derived membranes

**Fig. 3.**
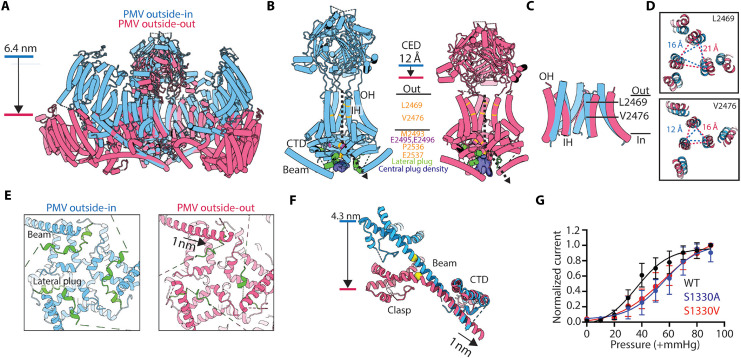
Conformational differences between curved and flat Piezo1 channels in PMVs. (**A**) Aligned structures of outside-in– (blue) and outside-out (red)–oriented Piezo1 from PMVs. (**B**) Isolated views of regions of Piezo1 involved in channel gating and ion conduction for the two Piezo1 structures are shown. Residues that form hydrophobic constrictions are color-coded as are residues identified as important for ion conduction. The lateral plug is highlighted, and the cryo-EM density for the central plug density is shown. The dashed arrow indicates the proposed ion conduction pathway for Piezo1. (**C**) Isolated view of the aligned OH and IH for the two Piezo1 structures. (**D**) Top-down views of two residue positions (L2469 and V2476) that form part of the hydrophobic constriction in the transmembrane pore of Piezo1. The distance between the Cα atoms of neighboring subunits is measured and annotated. (**E**) Isolated views of the cytosolic side of Piezo1 at the threefold axis. The beam helix and lateral plug (green) are annotated. The 1-nm sliding of the beam helix toward the central threefold axis appears to displace the lateral plug in the outside-out–oriented Piezo1 structure. (**F**) Isolated view of the Piezo1 beam helix, clasp, and CTD region from the aligned outside-out and outside-in PMV structures. Serine residue 1330, positioned proximal to a helical kink in the outside-out PMV Piezo1 structure, is depicted as yellow spheres in the two structures. (**G**) Outside-out pressure clamp recordings of cells overexpressing wild type (WT), S1330A, or S1330V Piezo1. Currents were held at +60 mV and pressurized from 0 to −90 mmHg. Peak currents at each pressure step were normalized to the peak current at −90 mmHg, and the data points were fit to a sigmoidal curve. *P*_50_ values are 36 ± 5 mmHg (WT), 56 ± 6 mmHg (S1330A), and 55 ± 6 mmHg (S1330V). *N* = 5 for all conditions.

Figure 3A shows the outside-in and outside-out models of Piezo1 superimposed. As shown previously, Piezo1’s shape is highly dependent on membrane bending forces (*33*, *34*). The outside-in structure is curved. This is because the channel tends to follow the intrinsic curvature of the vesicle. But, we know that Piezo1 does not conform perfectly to the vesicle shape, which is why outside-in Piezo1 vesicles are tear drop shaped rather than spherical (Fig. 2A) (*33*, *34*). The shape of the Piezo1 vesicle reflects the energy minimum condition reached when forces between the vesicle membrane and Piezo1 (including the lipid membrane between its three protein arms, called the Piezo dome) exactly counterbalance each other. Consequently, Piezo1 in the outside-in orientation is more curved than it would be in a planar membrane, and the vesicle deviates from a sphere. Similarly, the shape adopted by Piezo1 in an outside-out orientation must also reflect an energy minimum configuration. In this case, we observe a flat, disk-shaped Piezo1 channel and a vesicle that is distorted toward an oblate spheroid (Fig. 2A).

What local conformational changes occur inside the Piezo1 protein when it goes from highly curved to flat? In Fig. 3B, we show isolated structural elements thought to be important for channel gating and forming the ion conduction pore of Piezo1. These elements include the C-terminal extracellular domain (CED), outer and inner-helices (OH and IH) that form the transmembrane pore, and the C-terminal domain (CTD) that forms the intracellular region of the Piezo1 pore (*19*, *35*, *36*). In the flattened conformation relative to the curved, the CED is displaced ~12 Å toward the IH and OH (Fig. 3B and movie S1). This conformational change is notable because cysteine cross-links expected to limit the mobility of the CED inhibit mechanical gating (*37*). In addition, the OH and IH, which are attached directly to the CED via short linkers, are expanded in the flattened structure so that the pore’s extracellular vestibule is dilated (Fig. 3, B and C). Further into the membrane along the channel’s threefold axis the distance between Cα atoms of amino acids L2469 and V2476 are increased by ~5 and ~4 Å, respectively, in the flattened conformation (Fig. 3C). These specific amino acids have been identified through mutagenesis studies as forming a hydrophobic gate (*36*).

Numerous mutations have identified amino acids that affect ion conduction (*19*, *36*, *38*, *39*) and define what is thought to be the ion conduction pore (Fig. 3B). The resolution of our structures is too low to define the pore’s chemistry, but conformational differences along the pore are evident when comparing the curved and flat structures. In addition to the changes already mentioned involving the CED and dilation along the threefold axis, we also observe differences near the cytoplasmic opening (Fig. 3E). At the cytosolic end of the threefold axis narrow constrictions are formed by residues M2493, P2536, and E2537, which would prevent ion conduction (Fig. 3B). Instead, structural and mutagenesis studies support the idea that the pore trifurcates laterally into portals as it approaches the cytoplasm. In particular, the mutation of two highly conserved glutamate residues, E2495 and E2496, which line the lateral portal, alters ion permeability and conductance (*38*). In all previous structural studies, whether curved or flattened, the lateral portals are occluded by the lateral plug (*5*–*8*). The lateral plug is well-resolved in the curved structure, but in the flattened structure, the associated cryo-EM density appears fragmented and displaced, leading to the appearance of a cavity that might permit ion conduction (Fig. 3E and fig. S8).

In addition to the structural changes that appear to open the pore in the flat structure, we highlight in Fig. 3F structural changes that occur in what is called the beam helix, which projects along the cytoplasmic surface of each of the three Piezo1 arms. Flattening necessitates a kinking of the beam helix near S1330, whose role appears to complete a hydrogen bond in the kinked conformation. In support of this conclusion, when the serine is mutated to negate its hydrogen bonding capacity, mechanical gating is altered, requiring greater force to open the channel (Fig. 3G and fig. S9). The flattening of Piezo also causes a rigid body displacement of the beam helix toward the central axis of the channel, associated with the above-described conformational changes surrounding the pore (Fig. 3, E and F).

### Inferences on the elasticity of Piezo1 in cell-derived membranes

**Fig. 4.**
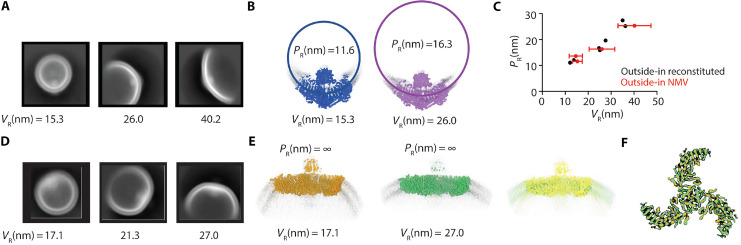
Piezo1 shape change analysis in PMVs of different size. (**A**) Example images of three populations of 2D classes generated by classification of aligned Piezo1 particles after extraction with a 60-nm box size. The mean vesicle radius, *V*_*R*_, is given below each image. The smallest vesicle class is the same as shown in Fig. 2 for outside-in oriented Piezo1. (**B**) Two example single particle cryo-EM maps of Piezo1 from different vesicle size classes are shown in blue and purple. A circle is fit to the mid-plane of the Piezo1 transmembrane helices to estimate the Piezo1 radius of curvature (*P*_*R*_), which is annotated along with the mean vesicle radius the particles came from. (**C**) *P*_*R*_ is plotted as a function of *V*_*R*_ for four classes of outside-in PMV Piezo1 structures, where *V*_*R*_ is the mean value derived from measurements of 30 vesicles for each class of particles, and the error bar represents the SD. Overlaid are previously published values from tomographic analysis of Piezo1 reconstituted into liposomes in the outside-in orientation (*33*). (**D**) As in (A), but here example images are given for Piezo1 particles from the outside-out PMV dataset after extraction with an 80-nm box size. (**E**) Two example single-particle cryo-EM maps of Piezo1 from the smallest and largest vesicle size classes in the outside-out PMV dataset are given in orange and green. On the right, an overlay of these two maps shows that the two structures are essentially identical. (**F**) A top-down view of the two outside-out Piezo1 structures as in (E), showing identical conformations.

If we look closely at the shape of Piezo1 in vesicles of different sizes, we observe that in larger vesicles, Piezo1 is less curved, that is, has a larger radius of curvature, *R*_*p*_ (Fig. 4, A and B). In a previous study, we documented the relationship between *R*_*p*_ and vesicle radius *R*_*v*_ by analyzing tomograms of individual Piezo1 vesicles made using reconstituted 1-palmitoyl-2-oleoyl-glycero-3-phosphocholine (POPC)/1,2-dioleoyl-sn-glycero-3-phospho-L-serine (DOPS)/cholesterol (8:1:1) lipids (Fig. 4C, black symbols) (*33*, *34*). In the present study using cell-derived membranes, after the alignment of Piezo1 proteins in cryo-EM images to a consensus structure, proteins were reextracted with a larger box size and two-dimensional (2D) classification was performed. In this second step, 2D classification is driven by the vesicle shape, permitting us to separate channels into classes based on vesicle size (Fig. 4, A and B for outside-in Piezo vesicles and Fig. 4, C and D for outside-out Piezo vesicles). After making 3D reconstructions of Piezo1 from each individual vesicle size class, the Piezo1 radius of curvature for each class was approximated by fitting a circle to the middle of the channel’s density using side views, as shown for outside-in Piezo1 vesicles (Fig. 4B). Figure 4C shows *R*_p_ graphed as a function *R*_*v*_ taken from the four different vesicle sizes. The graph also shows analogous data from the tomographic analysis of outside-in Piezo1 channels in POPC/DOPS/cholesterol vesicles (Fig. 4C, black symbols). The data follow a very similar trend for vesicles comprising POPC/DOPS/cholesterol and cell-derived lipids. From this correspondence, we infer that the elastic properties of Piezo vesicles over the range of outside-in vesicle sizes analyzed here are similar in both the defined and cell-derived lipid compositions. Because the bending modulus of these two lipid membranes is likely similar (*40*), the bending modulus of the Piezo dome must also be similar in both.

The outside-out cell-derived Piezo1 vesicles approximate oblate spheroids with a flat Piezo1 channel (Fig. 4D). Using the vesicle size classification approach described above, we find that outside-out Piezo1 channels adopt the same flat shape in vesicles with *R*_*v*_ = 27 nm and with *R*_*v*_ = 17 nm. From this observation, we conclude that once Piezo1 reaches a certain degree of flattening, it no longer changes its shape when a greater flattening force is applied. To clarify this statement, recall that when Piezo1 is inserted into a vesicle in the outside-in orientation, the vesicle curvature squeezes Piezo1 into a conformation that is more highly curved than it would be in a planar membrane (*33*, *34*). In the outside-out orientation, because the vesicle membrane runs opposite to Piezo1’s intrinsic curvature, the vesicle membrane applies a flattening force onto Piezo1 that is more extreme in smaller vesicles. The force applied by an *R*_*v*_ = 27-nm vesicles is sufficient to maximally flatten Piezo1. We tried to image larger outside-out Piezo1 vesicles to estimate the elastic properties of Piezo1 as the completely flat shape is approached but, for technical reasons, were unable to do this.

### Cell-derived lipids render a unique conformation of the flattened Piezo1 channel

**Fig. 5.**
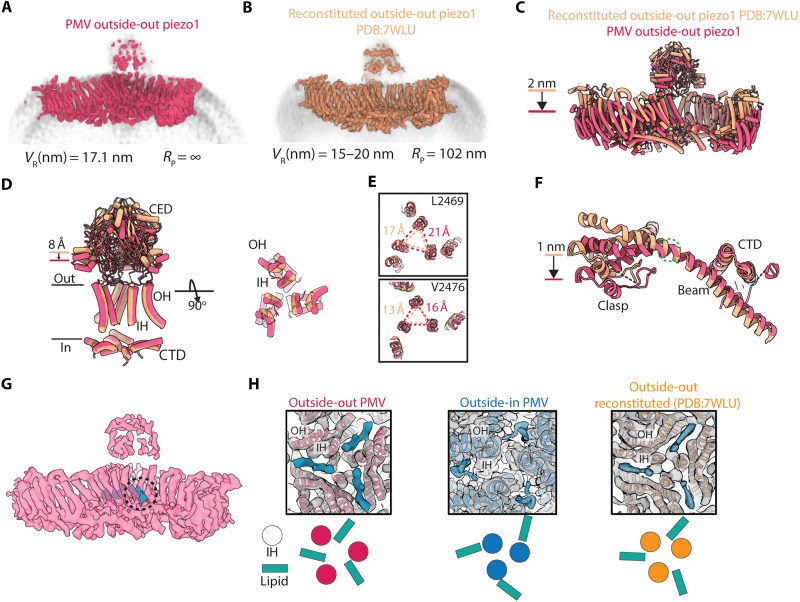
A cell membrane–derived lipid supports mechanical activation of Piezo1. (**A**) Left: Cryo-EM map of outside-oriented Piezo1 PMV particles with a mean radius of 17.1 nm. The membrane is contoured in gray, and the Piezo1 radius of curvature is labeled above. (**B**) Left: as in (A), but here a Piezo1 structure determined by others in reconstituted liposomes (*8*). (**C**) Aligned structures of the outside-out PMV (red) and reconstituted (orange) Piezo1 structures. The major displacement of the transmembrane arms is highlighted. (**D**) Left: Regions of Piezo1 involved in pore gating are aligned for the outside-out PMV and reconstituted Piezo1 structures. The downward displacement of the CED as Piezo1 flattens is labeled. Right: The pore-lining helices are shown after a 90^°^ rotation. (**E**) Two residue positions (L2469 and V2476) that form part of the hydrophobic constriction in the transmembrane pore region of Piezo1 are highlighted, and the distances between their Cα backbone residues are measured and annotated. (**F**) Isolated view of the Piezo1 beam helix, clasp, and CTD region from the outside-out PMV and reconstituted structures are shown. Serine residue 1330, positioned proximal to a helical kink in the outside-out PMV Piezo1 structure, is highlighted by a dashed green circle. (**G**) Cryo-EM map of Piezo1 from the outside-out PMV dataset (red) with an unmodeled extra density highlighted in blue. (**H**) Top-down views of the Piezo1 transmembrane pore, highlighting the location of additional density relative to the IH and OH pore helices. Below each annotated structure is a schematic illustrating the relative position of the putative cofactor to the IH.

We next compared the outside-out Piezo1 structure from cell-derived lipids in this study to a previously determined structure of Piezo1 reconstituted in an outside-out orientation in soy PC liposomes (Fig. 5) (*8*). Recall first that the functional data in Fig. 1 show that Piezo1 is functional in cell-derived membranes and not functional in soy PC membranes. Cryo-EM maps from similar sized vesicles show that Piezo1 remains curved (*R*_*p*_ about 100 nm) in PC membranes (Fig. 5B) and is completely flat (R_*p*_ → ∞) in cell-derived membranes (Fig. 5A). It is as if something about the soy PC lipid environment prevents, or fails to facilitate, complete flattening of Piezo1.

At the level of protein structure, complete flattening in cell-derived membranes compared to soy PC is associated with about a 2-nm displacement of the visible ends of the transmembrane arms (Fig. 5C). We summarize other conformational differences in Fig. 5 (C to F) this way: To a first approximation, similar conformational changes occur in soy PC but to a lesser extent. Also, our functional data suggest that the completion of this transition may account for why Piezo1 is functional in cell-derived membranes but not in soy PC or POPC:DOPS:cholesterol membranes. Together, these observations raise the question, what permits Piezo1 to complete the conformational transition in cell-derived membranes? Based on the data in Fig. 4C and previous studies on the dependence of the membrane bending elastic modulus on lipid composition (*33*, *34*), we think the structural differences are unlikely due to differences in the elastic properties of the membranes.

One possible explanation for the structural and functional differences is that cell-derived membranes supply an essential ingredient. In our outside-out PMV cryo-EM maps, we observe a prominent density that approaches the channel laterally from the inner leaflet of the bilayer, intercalating into the transmembrane pore between two adjacent IHs (Fig. 5, G and H). The shape of the density is consistent with a lipid headgroup and two branched acyl chains, but the density is not well enough resolved to assign a specific lipid. An electrostatic representation of the Piezo1 surface in this region reveals an electropositive region that appears to cup one end of the density, consistent with a binding site for a phospholipid headgroup (fig. S10). Several conserved lysine residues are near this putative lipid binding site (fig. S10), including K2182-K2185, which were identified as forming a potential phosphoinositide interaction site by molecular dynamics studies (*41*). The mutation of all four of these lysines to alanine, asparagine, or aspartate caused a shift toward larger pressures in the pressure-dependent activation threshold of Piezo1 in two separate studies (*41*, *42*), supporting a potential role of this region to Piezo1 gating.

In structures of Piezo1 determined in detergent micelles or after reconstitution into liposomes, density consistent with a “pore lipid” is often observed at a site close to the binding site we observe (Fig. 5H and fig. S10) (*8*, *43*). In all structures determined of Piezo1 in the curved, closed channel conformation, this putative lipid density resides outside the pore lining IHs, occluded from accessing the central pore, suggesting that pore expansion may be required for intercalation of the lipid between the IHs. In the partially flattened structure of reconstituted Piezo1 in the outside-out configuration, the lipid density resides just outside the pore, possibly reflecting a weaker binding interaction at this site compared to the lipid observed in our PMV structure. Notably, this pore-proximal binding site is also where an auxiliary subunit of Piezo1 and Piezo2, myoD family inhibitor domain-containing protein (MDFIC) binds (fig. S10) (*44*, *45*). MDFIC binding converts Piezo channels to high-threshold, slow-inactivating mechanosensitive channels, but the molecular mechanism of this function remains unclear. Thus, this binding site appears to be an important nexus for Piezo channel regulation.

**Fig. 6.**
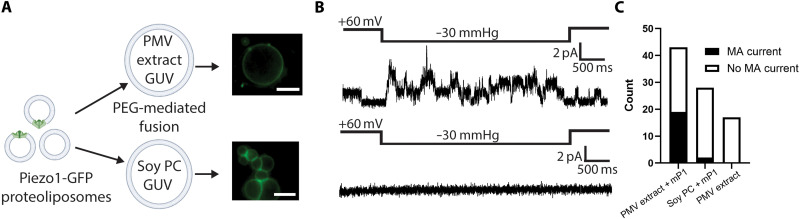
Mechanical activation of purified Piezo1 in lipids extracted from plasma membranes. (**A**) Left: Schematic illustrating PEG-mediated fusion of Piezo1-containing liposome into GUVs made from PMV lipid extract or soy PC lipids. Right: Fluorescence microscopy images showing successful incorporation of Piezo1-GFP into PMV (above) and soy PC (below) GUVs. Scale bars, 10 μm. (**B**) Example electrical recordings of patches excised from Piezo1-containing GUVs consisting of PMV (above) or soy PC (below) lipids. Patches were held at +60 mV and briefly pressurized to −30 mmHg to assess mechanically activated currents. (**C**) Survey of mechanically activated currents yielded by pressurizing stable patches excised from Piezo1 containing GUVs consisting of either PMV extract or soy PC lipids and PMV GUVs fused with empty liposomes. Patches were deemed stable if a gigaseal was maintained for over 1 min, and the mechanically activated (MA) current was defined as current that reversibly increased in amplitude in response to a pressure pulse.

To test our hypothesis that a lipid cofactor that is present in PMVs but not in soy PC vesicles enables mechanical activation of Piezo1, we performed the following experiment. First, soy PC liposomes containing Piezo1-GFP were fused into GUVs made from soy PC lipids or lipids extracted from HEK293 PMVs using PEG-8000 (polyethylene glycol, molecular weight 8000) (Fig. 6A and Materials and Methods). Patch-clamp recordings yielded mechanically activated currents for 19 of the 43 stable patches made from PMV extract GUVs (Fig. 6, B and C). By contrast, only 2 of the 28 stable patches made from soy PC GUVs yielded mechanically activated currents (Fig. 6, B and C). Mechanically activated currents were not observed in PMV extract GUVs fused with empty liposomes. Furthermore, the analysis of mechanically activated currents obtained from PMV extract GUVs revealed single-channel currents with conductance values consistent with Piezo1 function (fig. S11). The rare mechanically activated currents observed in soy PC lipids were in the background of noisy currents, where no discernible single channel conductance values could be measured (fig. S11), suggesting that these do not represent channel openings but instead transient bilayer instability, which can easily be observed when pressurizing membranes (*26*).

## DISCUSSION

Since the discovery of the Piezo channels, scientists have pursued an understanding of their force-dependent activation. In contrast to the prokaryotic mechanosensitive channels, which couple a large in-plane expansion associated with wide pore opening (*46*–*48*), Piezo1 channels activate at low lateral membrane tensions to form a cation-selective pore with a modest single-channel conductance (*1*, *49*). Piezo1 also exhibits complex gating behaviors including voltage-dependent inactivation, which varies markedly in different cell types (*1*, *37*, *44*, *50*). These properties likely enable the rich diversity of force-sensing processes Piezo1 is integral to. It is thus a worthwhile endeavor to understand how the Piezo channels are controlled by mechanical forces and other cues in living cells.

The first structures of Piezo1 determined in detergent, which revealed a highly curved, propeller-shaped trimeric channel, led to the membrane dome model (*6*). In this model, the deformation of the membrane bilayer by the Piezo1 transmembrane arms imbues the channel with its high-tension sensitivity, as flattening would be associated with a very large in-plane area expansion of the membrane dome, thus coupling Piezo’s shape through the product tension times area expansion. Piezo’s shape would have to be conformationally coupled to a more modest expansion of the channel’s pore, allowing the cation selectivity of Piezo1 we observe. Further structural studies of Piezo1 reconstituted into liposomes or microscopic studies of Piezo1 in cell membranes revealed that it can flatten under force (*8*–*9*, *33*–*34*, *51*), and quantitative studies of the Piezo1 shape as a function of vesicle size estimated that in a planar bilayer, like a cell membrane under nominal tension, the Piezo1 dome adopts an intrinsic radius of curvature of about 40 nm (*34*).

While these studies have demonstrated that Piezo1 can change its curvature (*8*, *9*, *51*) and even provided a quantitative relationship between Piezo1 shape and force (*33*, *34*), how mechanical sensing is transduced to open the pore and what properties give rise to the different gating behaviors of the channel in different physiological contexts have remained unclear. Central to our remaining ignorance is the fact that structural studies have been conducted under conditions that do not recapitulate force-dependent ion channel activity. Here, using single-particle cryo-EM analysis of Piezo1 in cell-derived vesicles, where we show that ion channel function is maintained, we describe conformational changes that can explain the activation of Piezo1 under force. Our most important observation is, in cell-derived membranes, Piezo1 can flatten to a degree, with pore-associated conformational changes, not seen previously in compositionally defined lipid membranes under otherwise similar conditions. These conformational changes are summarized in movie S1.

Recently, other studies have leveraged the mutagenesis of Piezo1 to examine the channel’s gating mechanism (*39*). The mutation of a serine residue to a glutamate (S2472E) within the transmembrane pore increased basal whole-cell poking currents of Piezo1 and slowed channel inactivation. One structure of Piezo1-S2472E in detergent was proposed to represent an intermediate conformation of channel opening. Based on alignments to our flattened structure, we observe that the mutation has likely disrupted the conformation of the pore, uncoupling the force- dependency of Piezo1 from conformational changes of the gating apparatus (fig. S12). While the pore-lining helices have expanded in the mutant structure, the conformation is distinct from what we observe in the flattened structure here and is not coupled to changes at the cytosolic gating apparatus (fig. S12).

What enables the fully flattened Piezo1 channel conformation we observe in cell-derived membranes? Our analyses suggest that a cofactor present in cell membranes and absent in previous reconstituted studies is necessary for complete flattening and the associated conformational changes to the Piezo1 gating apparatus we observe. The extra density we observe in the flattened structure is a good candidate for such a cofactor, and its properties are most consistent with a lipid molecule. Densities consistent with lipids have been observed adjacent to this binding site in multiple cryo-EM structures of Piezo1 in detergent and liposomes (*8*, *43*). In these structures, the channel remains closed, and the density does not intercalate between the pore-lining IHs. Thus, it is reasonable to hypothesize that lipids that do not support Piezo1 activation can occupy this binding site in purifications of Piezo1, reminiscent of how detergent molecules often occupy regulatory lipid-binding sites in cryo-EM structures of membrane proteins. This would be consistent with the absence of mechanical activation of purified Piezo1 reconstituted into soy PC liposomes, even under large, applied pressures.

In contrast, we show that the reversible mechanical activation of Piezo1 can often be observed after the channel is incorporated into vesicles made from HEK293 cell plasma membrane lipids. This observation is consistent with a lipid present in mammalian plasma membranes enabling pressure-dependent activation of Piezo1. Both ceramide and phosphatidylinositol 4,5-bisphosphate (PIP_2_) have been shown to be functionally important for Piezo1 activation (*50*, *52*), and molecular dynamics simulations implicate an abundance of interaction sites to the channel (*53*, *54*). More work will be needed to identify and validate the role of this cofactor. The idea that Piezo1 is primarily mechanosensitive but requires a cofactor for activation is very reminiscent of some other ion channels that exhibit multiple modes of gating. The Kv7.1 K^+^ channel is a good example (*55*, *56*). Its opening is voltage dependent but requires PIP_2_ to open. Membrane depolarization alone is insufficient.

**Fig. 7.**
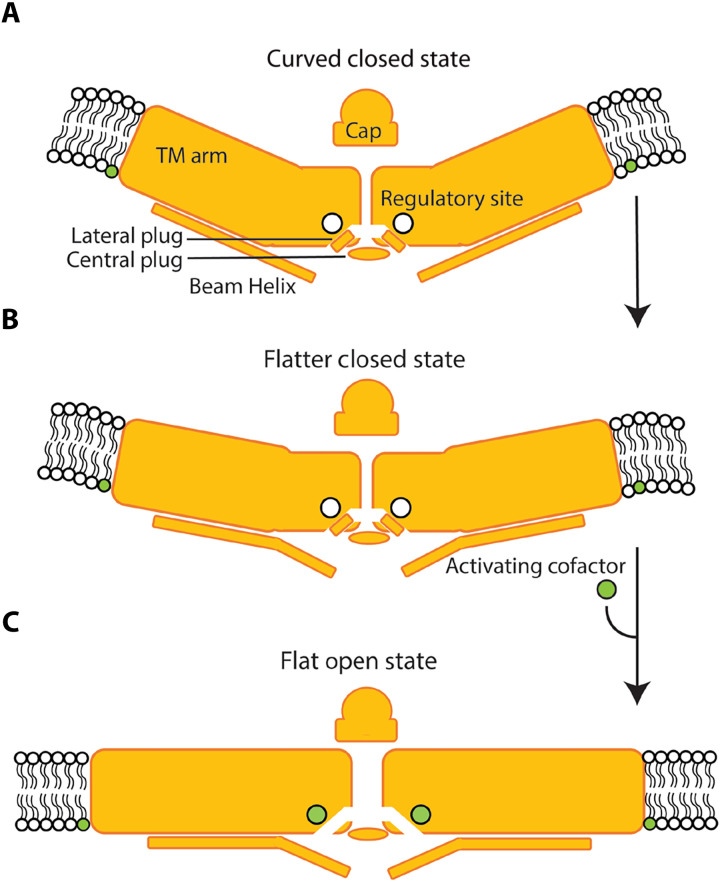
Proposed mechanism for multimodal gating of Piezo1. (**A**) Under resting conditions in an approximately planar cell membrane, the transmembrane (TM) arms of Piezo1 adopt a slightly curved conformation with an intrinsic radius of curvature of ~42 nm. (**B**) With increasing membrane tension, the TM arms of Piezo1 begin to flatten, supported by the beam helix. Without occupancy of the regulatory site the channel pore remains closed. (**C**) At some given mechanical force, in combination with binding of the activating cofactor at the regulatory site, Piezo1 flattens fully into an activated conformation, associated with displacement of the lateral plug, enabling ion conduction.

A simple schematic for a multimodal gating of Piezo1 is shown in Fig. 7. To achieve the activated conformation both mechanical force and the cofactor are required. In the depiction, the two events—mechanical force and the cofactor binding—occur sequentially, but this point needs further study. The requirement of two stimuli might rationalize the variable, environment-dependent properties of Piezo1 function, such as channel inactivation, observed in different cell types and under different conditions (*1*, *37*, *44*, *50*). There is evidence for both lipid-mediated (*30*, *50*) and protein-mediated modulation of Piezo1 function (*44*, *57*), with some described mechanisms being cell-type specific (*44*, *50*). Most recently, the transcriptional regulator, MDFIC, which abrogates channel inactivation, was shown to bind at the same regulatory site postulated in the present study (fig. S10). So far, all structures of the Piezo-MDFIC complex have been determined in detergent micelles, where the Piezo channel is in the curved, closed conformation. The physical basis for MDFIC’s modulation of Piezo channel function is still unclear, but it is reasonable to hypothesize that under membrane tension, as Piezo1 flattens and the pore-lining helices expand, MDFIC occupies the same binding site, intercalated between the IHs, that we observe occupied by a putative lipid in our flattened PMV structure. Through this mechanism, it could stabilize the open channel pore conformation, slowing channel inactivation as observed in electrical recordings (*44*, *45*). Overall, this binding site could serve as a nexus for cues additional to mechanical forces that regulate Piezo1 function, enabling the rich diversity of force-sensing processes Piezo1 has been ascribed to.

## MATERIALS AND METHODS

### Cell culture and transfection

PIEZO1^−/−^ HEK293T cells were generated previously by CRISPR-Cas9 technology in the laboratory (*18*) and were cultured in Dulbecco’s modified Eagle’s medium (DMEM) (Gibco) supplemented with 10% fetal bovine serum. *Spodoptera frugiperda* Sf9 cells (American Type Culture Collection) were cultured in Sf-900 II SFM medium supplemented with penicillin (100 U/ml) and streptomycin (100 U/ml) at 27°C. Expi293F cells (Thermo Fisher Scientific) were cultured in Expi293 expression medium (Thermo Fisher Scientific)

### Protein expression

Piezo1 was heterologously expressed in Expi293F cells using the BacMam method as previously described (*6*, *58*). Bacmids were generated by transforming the mPiezo1-ALFA_2420_-HRV3C-GFP construct into *Escherichia coli* DH10Bac cells. Baculoviruses were then produced by transfecting Sf9 cell with the bacmid using Cellfectin II (Invitrogen). After two rounds of amplification, baculoviruses were used for cell transduction. Expi293F cell suspension cultures were grown at 37°C to a density of ~3 × 10^6^ cells/ml and infected with 10% (v/v) baculovirus. After 16 hours, 10 mM sodium butyrate was supplemented, and the temperature was shifted to 30°C. Cells were harvested ~48 hours later. For protein purification and reconstitution, cell pellets were flash-frozen in liquid nitrogen and used later. For NEM-induced membrane vesicle (PMV) isolation, cells were used immediately without freezing.

### Purification of Piezo1 in detergent

Cell pellet from 2 liters of Expi293F cells expressing mPiezo1-ALFA_2420_-HRV3C-GFP was resuspended in 200 ml of buffer containing 150 mM NaCl, 20 mM tris-buffered saline (pH 8; TBS), and a protease inhibitor mix containing pepstatin A (0.1 μg/ml), leupeptin (1 μg/ml), aprotinin (1 μg/ml), 1 mM benzamidine, 4-(2-aminoethyl) benzenesulfonyl fluoride hydrochloride (AEBSF; 0.1 mg/ml), and 1 mM phenylmethylsulfonyl fluoride hydrochloride (PMSF). Resuspended cells on ice were lysed by sonication with a probe sonication (1/2″ tip with the Branson102-C converter) at 40% power for 6 × 30-s pulses, with 30-s delays between. After a 5000*g* spin for 15 min, the supernatant was ultracentrifuged at 100,000*g* at 4°C for 40 min. The resultant membrane pellet was resuspended in TBS (5 ml per pellet) and dounce homogenized for ~20 strokes. The membrane suspension was solubilized in TBS buffer containing 2% C12E10 and protease inhibitors for 90 min at 4°C. The solubilized suspension was then ultracentrifuged at 100,000*g* at 4°C for 40 min, and the resultant supernatant was incubated with 1 ml of ALFA Selector CE resin (NanoTag), preequilibrated with TBS buffer containing 0.025% C12E10 [size exclusion chromatography (SEC) buffer], at 4°C overnight.

The following day, the ALFA selector CE resin was batch-washed twice with ~20 ml of SEC buffer. The resin was then loaded onto a gravity column and washed with another 15 ml of SEC buffer. Protein was then eluted in 10 ml of SEC buffer containing 0.2 mM ALFA peptide (NanoTag) by incubation at 4°C for 2 hours. Eluted protein was concentrated to ~500 μl using an Amicon 4 ml concentrator (molecular weight cutoff of 30 kDa). After a 5000*g* spin for 5 min at 4°C to pellet aggregate, the supernatant was further purified on a Superose-6 size exclusion column (10/300 GL) in SEC buffer at 4°C. The peak fractions corresponding to the trimeric mPiezo1 were concentrated to ~2 mg/ml using a 2-ml Amicon concentrator (molecular weight cutoff of 30 kDa) to be used for reconstitution into liposomes.

### Reconstitution of Piezo1 into liposomes

Soy PC (Avanti) lipids were resuspended in chloroform at a concentration of 20 mg/ml and dried to a thin film under argon. The lipid film was further dried overnight in a room temperature (RT) vacuum desiccator and then resuspended at a concentration of 10 mg/ml by mixing with TBS buffer. SUVs were formed by bath sonication of 4 mg of lipids at RT until the solution was mostly transparent, and the suspension was solubilized by the addition of 2% *n*-decyl-ß-d-maltoside (DM) (Avanti) for 30 min at RT. Solubilized lipids were mixed with concentrated Piezo1 at protein/lipid ratios of 1:50 and 1:500 for 1 hour at 4°C. Detergent was removed using adsorbent Bio-Beads SM-2 Resin (Bio-Rad) by adding 20 mg of a 50% (w/v) Bio-Beads slurry in TBS buffer and rotating at 4°C overnight. The proteoliposomes were then concentrated ~5× in a 500-μl Amicon spin concentrator (molecular weight cutoff of 100 kDa). The concentrated proteoliposomes were then flash-frozen in liquid nitrogen to use later.

### Extraction of lipids from PMVs

One liter of Expi293 F cells was grown to a density of ~4 to 6 million cells/ml. The cell pellet was resuspended in 100 ml of GPMV buffer containing 140 mM NaCl, 5 mM KCl, 10 mM Na-Hepes, 2 mM CaCl_2_, and 7.5 mM NM at pH 7.4. The cell suspension was incubated in baffled flasks at 37°C, shaking at 130 rpm for 2 hours. After incubation, the flasks were shook vigorously by hand for ~20 s to release more vesicles, before spinning at 3000*g* at 4°C for 10 min to pellet cells and GPMVs. The remaining SPMVs were ultracentrifuged at 100,000*g* at 4°C for 30 min in a Ti70 rotor. The membrane pellets were resuspended in 4 ml of water and dounce homogenized. In glass tubes, 2 ml of lipid suspension was mixed with 4 ml of methanol and 2 ml of chloroform. After mixing for 10 min, 2 ml of chloroform and 2 ml of water were added. The solution was mixed by inversion and then left standing in RT for 3 hours to phase separate. The solubilized lipid mix was aspirated from the bottom layer and filtered through an Acrodisc LC PVDF 0.2 μM filter. The solution was dried down with argon, washed with 1 ml of pentane, and then dried down with argon again before being placed under vacuum overnight.

### Fusion of Piezo1 liposomes into preformed GUVs

Piezo1-GFP was reconstituted into soy PC liposomes as described above except the protein/lipid ratio used was 1:10. GUVs were preformed by spotting 5 μl of solubilized lipids (10 mg/ml) in chloroform onto an indium tin oxide (ITO)–coated glass slide (Sigma-Aldrich). An electroformation chamber was assembled with two ITO slides sandwiched over silicone isolators (13 mm in diameter and 2.4 mm in depth, Grace Bio-labs). Each ITO slide contained one strip of copper conductive tape at one end. The electroformation chamber was placed under vacuum for 3 hours or overnight to dry down the lipids. To form GUVs, solution containing 200 mM sucrose, 10 mM KCl, and 10 mM Hepes 7.4 was added to the chamber before connection to a Velleman function generator. Electroformation was performed with a protocol starting at 0.6 V, 20-Hz frequency, and ramping to 5.7 V over 30 min, holding at these values for 90 min before reducing the frequency to 5 Hz for 30 min to bud off GUVs.

To fuse GUVs with Piezo1-containing proteolposomes, 50 μl of GUVs was mixed with 5 μl of proteoliposomes and 45 μl of fusion buffer [70 mM glucose, 50 mM KCl, 10 Hepes (pH 7.4)] and 1 mM CaCl_2_ and incubated at RT for 10 min. PEG-8000 (40% stock solution in fusion buffer) was added to a final concentration of 7.5%, and the solution was slowly mixed by inversion for 10 min at RT. The mixture was diluted fivefold in fusion buffer, and the GUVs were gently pelleted by a 600*g* spin. A 450 μl of solution was removed without disruption of the bottom layer, and the GUVs were diluted again fivefold in fusion buffer to lower the concentration of PEG-8000. After a 600*g* spin, GUVs could be aspirated from the bottom of the tube and used for electrophysiology experiments. Glass-bottomed dishes were precoated with a bovine serum albumin solution (2 mg/ml) to passivate the glass before addition of bath solution and fused GUVs.

### Electrophysiology

For recordings of Pizeo1 function from cells, 3 μg of plasmid was transfected into HEK293 cells at ~50 to 60% confluency using FuGENE HD transfection reagent following the manufacturer’s instructions (Promega). Recordings were made 24 to 48 hours posttransfection.

For patch clamp recordings of GPMVs, cells were first transfected with plasmid as described above. Following expression, DMEM was removed from the adherent HEK293 cells, which were then washed twice with GPMV buffer: 150 mM NaCl, 5 mM KCl, 20 mM Na-Hepes, and 2 mM CaCl_2_ (pH 7.4) before then being incubated with GPMV buffer + 7.5 mM NEM for 2 hours at 37°C. The solution was then aspirated using a 1000-μl pipette with the tip cut off and allowed to sit at RT for 10 min to pellet detached cells and free GPMVs. A 20 μl of GPMVs, aspirated from just above the cell pellet, was then added to bath solution in a glass-bottomed dish.

For patch clamp recordings of reconstituted Piezo1 by the dehydration/rehydration method, giant uinlamellar vesicles were formed from Piezo1-containing proteoliposomes. A 10 μl of proteoliposome solution was pipetted into ~6 spots on a glass-bottomed dish and dried for at least 6 hours at RT in a vacuum desiccator. A 50 μl of rehydration buffer containing 140 mM NaCl and 20 mM Na-Hepes (pH 7.4) was then added to the dried down spots, and the dish was placed into a 150 mm–by–25 mm dish containing wet Kimwipes and allowed to rehydrate overnight at 4°C. The following day, bath solution was added, and after 30 mins, the sample was ready for patch clamping.

For all electrophysiology experiments, unless stated otherwise, bath solution and pipette solution consisted of 140 mM NaCl and 20 mM Na-Hepes at pH 7.4. Imaging was performed on a Nikon eclipse DIC microscope at ×40 magnification. Pipettes of borosilicate glass (Sutter Instruments, BF150-86-10) were pulled to ~2- to 5-MΩ resistance with a micropipette puller (Sutter Instruments, P-97) and polished with a microforge (Narishige, MF-83). Recordings were obtained with an Axopatch 200B amplifier (Molecular Devices), filtered at 1 kHz, and digitized at 10 kHz (Digidata 1440A, Molecular Devices). A high-speed pressure clamp (ALA scientific) was used to form gigaseals and reversibly apply pressures to activate Piezo1 channels. For excised recordings from GPMVs and GUVs, if the vesicle remained attached after gigaseal formation and pulling away of the pipette, the vesicle would be lifted briefly to the air-water interface to remove the attached vesicle but leave an intact membrane patch inside the pipette.

### Piezo1 PMV preparation and purification

After expression of Piezo1-ALFA_2420_-GFP by baculovirus transduction as described above, 4 liters of live cells were harvested by centrifugation at 3000*g* for 10 min. The cell pellet was resuspended in 400 ml of GPMV buffer containing 140 mM NaCl, 5 mM KCl, 10 mM Na-Hepes, 2 mM CaCl_2_, and 7.5 mM NEM at pH 7.4. The cell suspension was incubated in baffled flasks at 37°C with shaking at 130 rpm for 2 hours. After incubation, the flasks were shook vigorously by hand for ~20 s to release more vesicles, before spinning at 3000*g* at 4°C for 10 min to pellet cells and GPMVs.

The supernatant was then supplemented with a protease inhibitor mix containing pepstatin A (0.1 μg/ml), leupeptin (1 μg/ml), aprotinin (1 μg/ml), 1 mM benzamidine, AEBSF (0.1 mg/ml), 1 mM PMSF, and 10% glycerol before sonication with a probe sonicator at 40% power for 30-s pulses, six times with 30-s breaks in between. The vesicles were spun at 3000*g* for 10 min to pellet aggregate before ultracentrifugation at 100,000*g* at 4°C for 30 min in a Ti70 rotor. The membrane pellet from every ~25-ml sample was triturated in ~1 ml of GPMV buffer supplemented with 10% glycerol. The resuspended vesicles were then sonicated in a bath sonicator (Branson M1800) for ~30 s. A centrifugation step at 3500*g* for 10 min was then performed to pellet the remaining aggregates. The following affinity purification steps then depended on whether we were isolating inside-out or outside-out Piezo1 PMVs.

For inside-out PMVs, the vesicles were incubated with 2 ml of GFP-Trap Magnetic Particles M-270 resin (Proteintech), preequilibrated with GPMV buffer, for 2 hours at RT. The resin was then separated from flowthrough solution using a DynaMag-2 magnet and washed with 10 ml of total GPMV buffer three times. The bound vesicles were then eluted by incubating with HRV-3C protease at a final concentration of ~0.014 mg/ml at RT for 2 hours. The eluted vesicles were concentrated in an Amicon 0.5 ml concentrator (molecular weight cutoff of 100 kDa) to an optical density at 280 (OD_280_) of ~2 to 4.

For outside-out PMVs, the vesicles were incubated with 2 ml of ALFA Selector PE resin (NanoTag) preequilibrated with GPMV buffer at 4°C overnight. The following day, the ALFA selector PE resin with bound vesicles was first batch-washed twice with ~20 ml of GPMV buffer and spun at 1000*g* at 4°C for 1 min to collect resin, followed by a second wash with 20 ml of GPMV buffer. The resin was then loaded onto gravity column and washed further with 20 ml of GPMV buffer. The vesicles were eluted with 10 ml of GPMV buffer supplemented with 0.2 mM ALFA peptide by incubating at RT for 1 hour. Because of the persistent occurrence of inside-out Piezo1 PMVs and Piezo1-containing broken membrane “fragments,” another purification step was included to sequester these contaminants. Eluted vesicles were incubated with 2 ml of GFP-Trap Magnetic Particles M-270 resin (Proteintech), preequilibrated with GPMV buffer, for 2 hours at RT. The supernatant was separated from the resin using a Dynamag-2 magnet, and the flow-through, containing mostly outside-out Piezo1 PMVs, was concentrated in an Amicon 0.5 ml concentrator (molecular weight cutoff of 100 kDa) to an OD_280_ of ~2 to 4.

### Grid preparation and data collection

Quantfoil R1.2/1.3 400 mesh holey carbon grids were glow-discharged for 22 s. Then, 3.5 μl of concentrated membrane vesicles was applied to freshly glow-discharged grids and left for 5 to 8 min at 22°C. The grids were blotted manually with a piece of filter paper before another 3.5 μl of sample was applied. The grids were blotted using a Vitrobot Mark IV with a blot force of 0 and blot time of 3 s after 20 s of incubation. The grids were flash-frozen in liquid ethane and stored in liquid nitrogen until data collection.

Both the inside-out and outside-out Piezo1 PMV datasets were collected on a 300-keV Titan Krios transmission electron microscope equipped with a cold-field emission gun and an energy filter (slid with 6 eV). For the inside-out PMV dataset, a total of 19,144 movies were recorded by a Falcon 4i camera with a physical pixel size of 0.94 Å and a target defocus value of −1.5 to −2.5 μm. The movies have 2142 internal frames and a total dose of 60 e^−^/ Å^2^. For the outside-out PMV dataset, a total of 16,060 movies were recorded by a Falcon 4i camera with a physical pixel size of 0.94 Å and a target efocus value of −1.5 to −2.5 μm. The movies have 1953 internal frames and a total dose of 60 e^−^/ Å^2^.

### Cryo-EM data processing

The data-processing workflow is detailed in figs. S2 and S4. Data processing was carried out using cryoSPARC v4 and RELION 4.0. The movies were gain-normalized and motion-corrected in CryoSPARC. Contrast transfer function parameters were estimated from the motion-corrected micrographs using the Patch CTF estimation tool. All subsequent processing was performed on motion-corrected micrographs with dose weighting.

For the outside-in PMV dataset (fig. S2), ~500 particles were first manually picked, separately selecting side views and top views of Piezo1. These sets of particles were then used to train a TOPAZ picking model, which was subsequently used to pick particles on approximately a fifth of the dataset. Good 2D classes were selected and used to retrain a TOPAZ picking model, and this was done iteratively to improve the picking model. A combined total of 529,310 particles were picked from the side and top views. The best side view 2D classes were picked, and an initial map was generated by ab initio reconstruction. This map was refined by homogenous refinement in C3 and used as a reference for heterogeneous refinement to sort for the well-aligned particles that were then refined in C3. Next, a seeded heterorefinement method was used to enrich for good particles and include top views. In brief, particles selected from good 2D classes of top views and side views were split into subgroups, combined with the well-aligned particles seed particles selected from previous steps, and hetereogeneous refinement was performed. For each round, the good classes were selected, the redundant particles were removed, and refinement was performed. To improve the density of the CED, the CED and central pore unit were masked out and locally refined in C3. To improve the density of the transmembrane arms, the particles were symmetry-expanded in C3, and one arm with the CED was masked out before local refinement was performed in C1. These focused maps were then combined in Phenix to generate the final reconstruction.

For the outside-out PMV dataset (fig. S4), ~500 particles were first manually picked and used to train a TOPAZ picking model, which was used to repick particles, and iterative rounds of this were performed to achieve the best TOPAZ picking model. From these particles, 2D classification was performed. Despite extensive efforts, we were unable to get good 2D classes of top-down views. From the best ~100,000 particles, hetereogenous refinement was performed using a 60-Å low-pass–filtered map of a previously determined structure of outside-out–oriented Piezo1 reconstituted into liposomes (EMD-32593) (*8*). Multiple rounds of heterorefinement were performed this way. The best-aligned particles and map were used for a seeded heterorefinement strategy, as discussed above, enriching the number of well-aligned particles approximately sixfold. After combining the good particles and removing redundante ones, C3 refinement was performed, and these particles were expanded by C3 symmetry. One transmembrane arm and CED were masked out, and RELION 3D classification was used without alignment in C1 to separate out the bad particles. The best particles then went through another round of 3D classification, this time with local angular searches, and the best particles were then imported into CryoSPARC, the duplicates were removed, and local refinement in C3 was used to achieve the final map.

### Model building and refinement

A structural model for the inside-out Piezo1 PMV was built by docking in the structure of Piezo1 in detergent micelle [Protein Data Bank (PDB) ID:6B3R] (*6*) and adjusted using the ISOLDE plugin (*59*) in Chimera X (*60*) or Coot (*61*), followed by real-space refinement in Phenix (*62*). The outside-out Piezo1 PMV structural model was built using a starting structure of Piezo1 reconstituted into liposomes (PDB ID: 7WLU) (*8*) that was fit into the map by rigid-body refinement in ISOLDE and Phenix. The quality of the final models was evaluated using the MolProbity plugin (*63*) in Phenix. Graphical representations of models and cryo-EM maps were prepared using ChimeraX. Map and structure statistics for the two structures are given in table S1.
