# Supplementary material for: Lipid composition and mechanical force underlie multi-modal regulation of Piezo1 gating
Source: Sci Adv. 2026 Jun 3;12(23):eaed7115. doi: 10.1126/sciadv.aed7115 (PMC13232553; doi:10.1126/sciadv.aed7115)
Supplement: Supplementary file 1 — Figs. S1 to S12 Tables S1 and S2 Legend for movie S1 [file sciadv.aed7115_sm.pdf]

Science Advances
AAAS

Supplementary Materials for

Lipid composition and mechanical force underlie multi-modal regulation of Piezo1 gating

George Vaisey and Roderick MacKinnon

Corresponding author: Roderick MacKinnon, mackinn@rockefeller.edu

Sci. Adv. **12**, eaed7115 (2026)
DOI: 10.1126/sciadv.aed7115

The PDF file includes:

Figs. S1 to S12
Tables S1 and S2
Legend for movie S1

Other Supplementary Material for this manuscript includes the following:

Movie S1

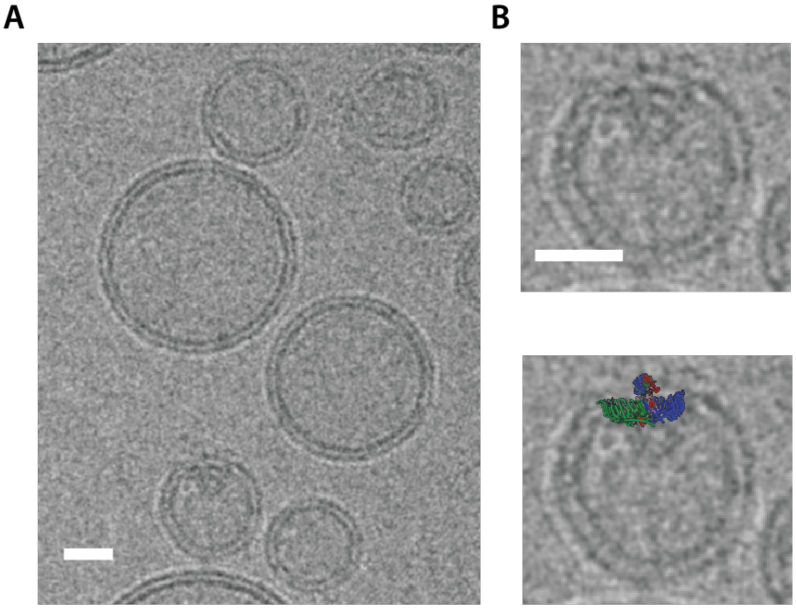

Fig. S1. Reconstitution of purified Piezo1 into soy PC liposomes. (A) Raw micrograph image from electron microscopy of Piezo1 reconstituted into soy PC liposomes. (B) Close-up of a liposome containing a Piezo1 channel with a structure of Piezo1 overlaid for clarification. Scale bar = 20 nm.

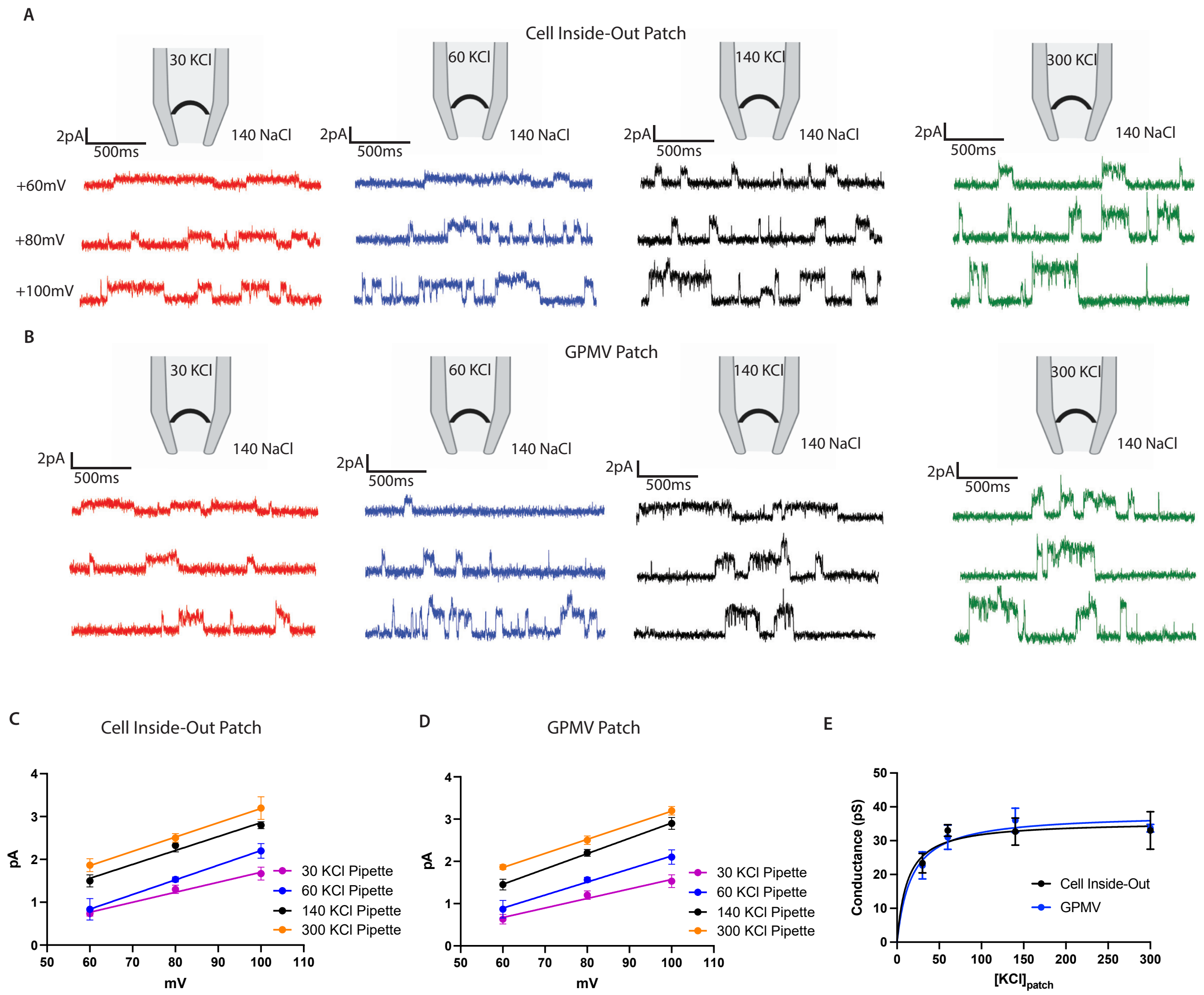

Fig. S2. Conductance measurements of Piezo1 in varying [KCl]. (A) Example recordings of inside-out patches excised from HEK293 cells overexpressing mPiezo1. A representative 2s of recordings held at different positive voltages from patches under increasing [KCl] are shown. (B) As in *A* but for patches excised from GPMVs. (C) Single channel current-voltage relationships are shown for inside-out patches under different [KCl] conditions, with error bars indicating the standard deviation (n=3). The data points were fit by a simple linear regression. (D) as in *C* but for GPMV patch measurements. (E) Piezo1 conductance as a function of increasing [KCl]. Slope measurements from *C* and *D* were used to determine conductance values. The mean and standard deviation are plotted from n=3 recordings at each [KCl] for cell inside-out and GPMV measurements.

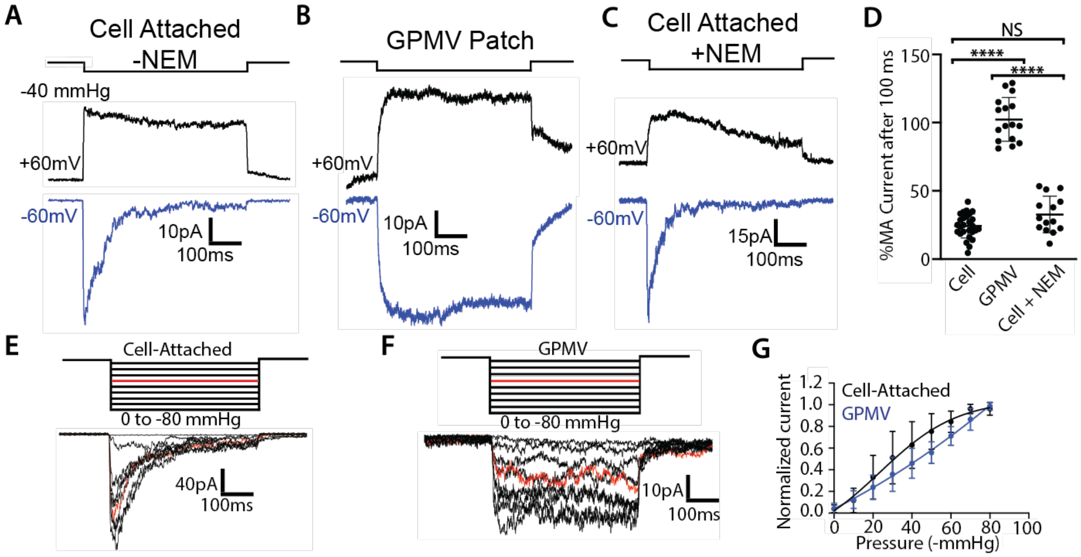

Fig. S3. Channel properties of Piezo1 in GPMVs. (A) Representative cell-attached recording from HEK293 cells overexpressing Piezo1 showing Piezo1 inactivation at physiologically negative voltages in the absence of NEM under applied pressure. (B) as in *A* but for GPMV patch recordings. (C) as in *A* but in for cell-attached recordings in the presence of 7.5 mM NEM in the bath solution. (D) Comparison of Piezo1 inactivation in cell-attached (n=27), GPMV (n= 16) and cell-attached in the presence of 7.5 mM NEM in the bath solution for one hour (n=14). Inactivation was quantified as the percentage of mechanically activated (MA) current remaining 100 ms into a -40mmHg pressure pulse at -60 mV. Statistical significance was valued by a one-way analysis of variance (ANOVA) test with Tukeys post hoc analysis, as indicated on the graph by asterisks where **** indicates p<0.0001 and NS indicates no statistically significant difference. (E) Representative cell-attached recording from HEK293 cells overexpressing Piezo1 showing pressure-dependent channel activation. The patch was held at 0mV before going to -60mV under increasing negative pressures from 0 to -80 mmHg. The current at -40 mmHg is highlighted in red. (F) As in *E* but for GPMV patch recordings. (G) Pressure-dependent channel activity was compared for cell-attached and GPMV recordings. Measurements from recordings as in *E* and *F* were used to plot current, normalized to the maximum current for each recording, at pressures from 0 to -80 mmHg. The mean normalized current at each pressure and standard deviation are plotted from n= 19 (cell-attached) and n=15 (GPMV) recordings. The datapoints were fit to a sigmoidal curve, yielding a P_{50} value of -23.6 ± 7.4 mmHg for cell-attached recordings. The currents did not saturate at -80 mmHg for GPMV recordings and therefore a P_{50} could not be determined.

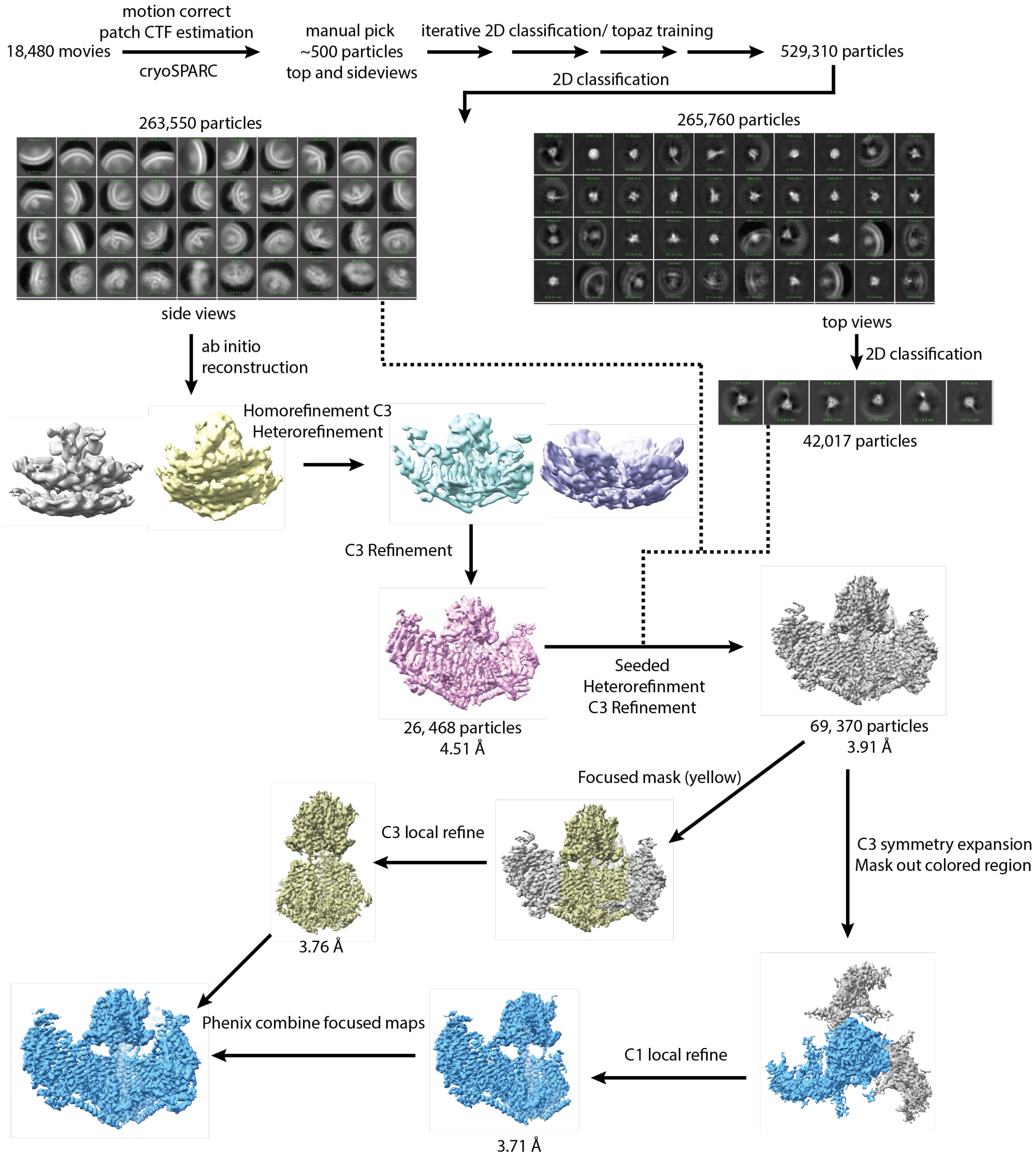

Fig. S4. Single particle cryo-EM processing workflow for the outside-in Piezo1 PMV dataset

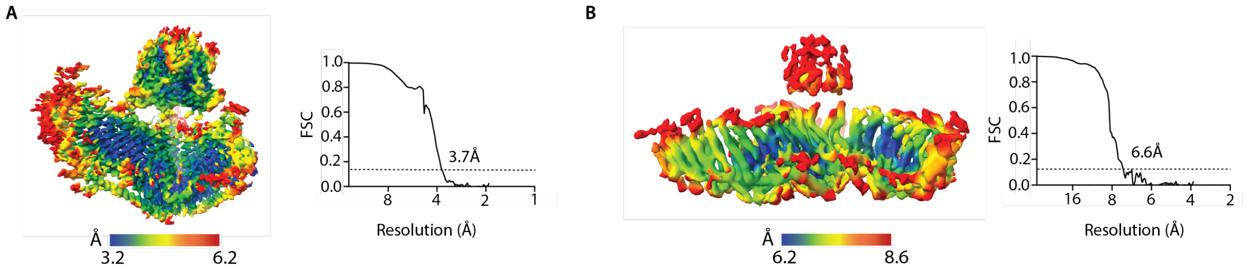

Fig. S5. Local resolution maps and FSC curves. The local resolution maps and FSC curvs are generated by CryoSPARC. **(A)** Outside-in Piezo1 PMV. **(B)** Outside-out Piezo1 PMV.

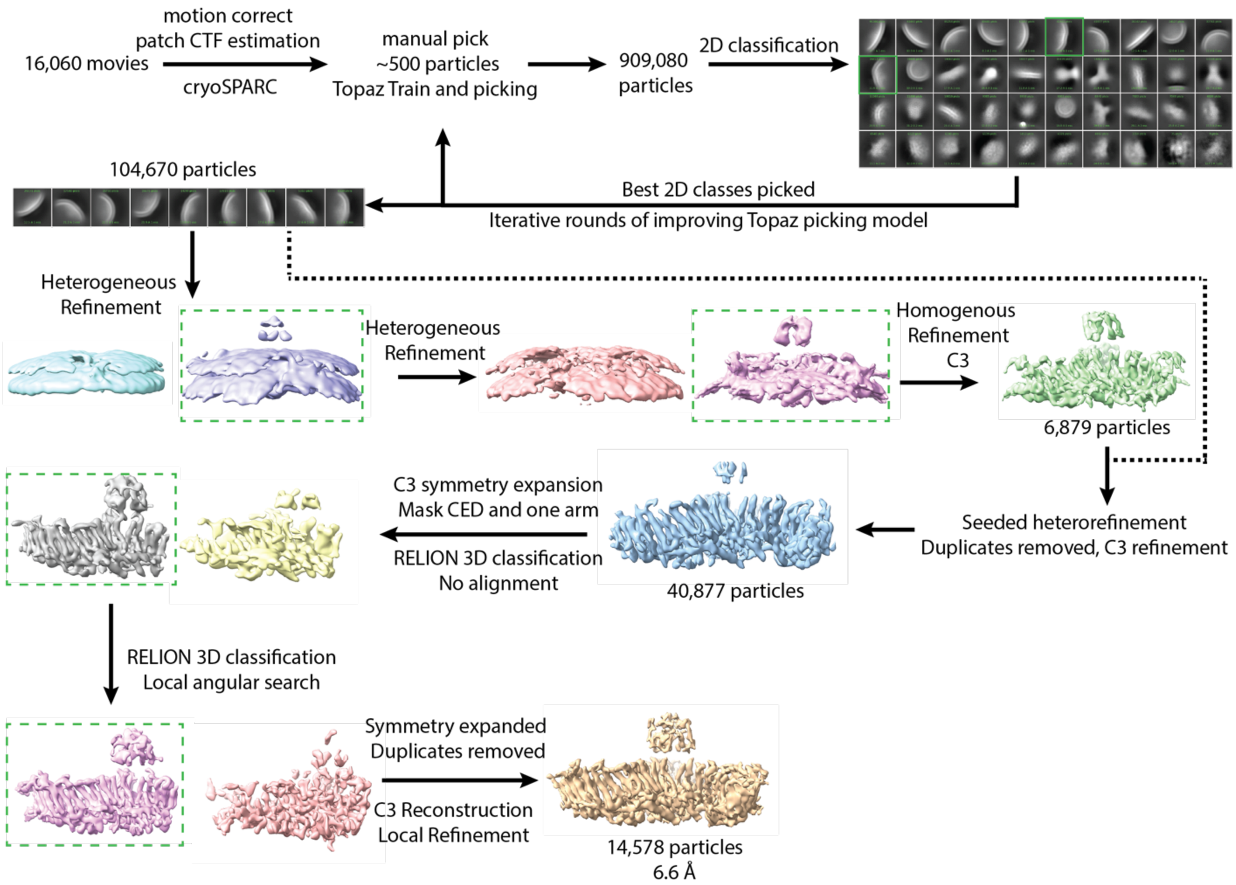

Fig. S6. Single particle cryo-EM processing workflow for the outside-out Piezo1 PMV dataset

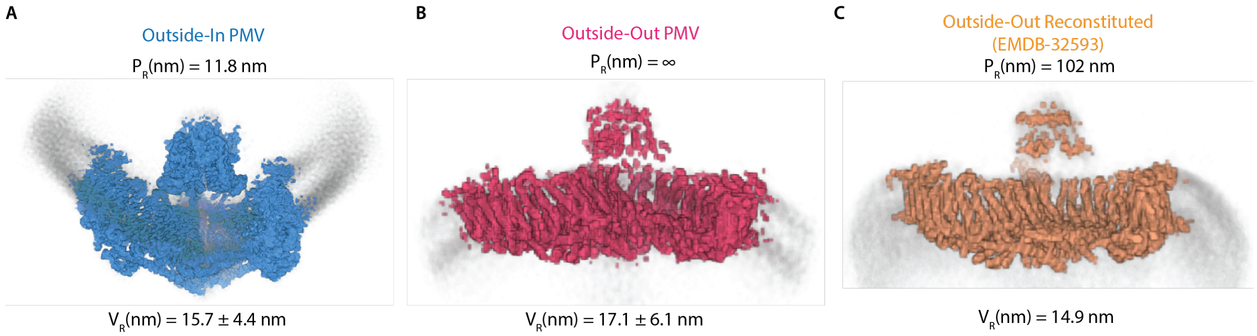

Fig. S7. Cryo-EM maps of Piezo1 in vesicles. (A) Cryo-EM map of outside-in oriented Piezo1 in PMVs with the membrane contoured in grey. The Piezo1 radius of curvature and the mean radius of the vesicles contributing to this structure are annotated. **(B)** as in *A* for the outside-out Piezo1 PMV structure. **(C)** As in *A* for a structure determined by others of Piezo1 reconstituted in the outside-out orientation in liposomes[8].

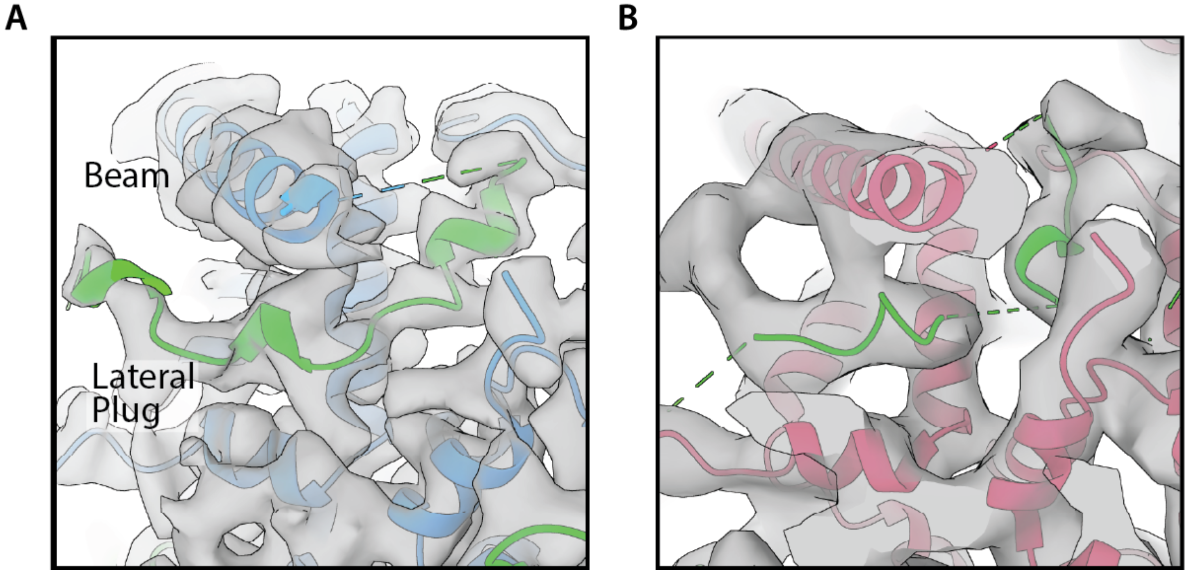

Fig. S8. Cryo-EM map of the Lateral Plug. Close-up view of the lateral plug structure (green) and cryo-EM map (gray) for the outside-in (A) and outside-out (B) PMV Piezo1 structures.

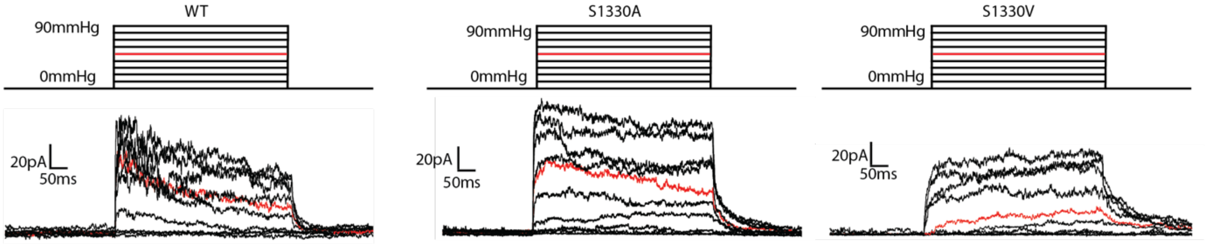

Fig. S9. Representative pressure-activated currents of WT Piezo1 compared to two mutations made at a serine residue in the beam helix. Outside-out patch clamp recordings were made from Piezo1 knockout HEK293 cells overexpressing either WT Piezo1 or one of the mutants S1330A or S1330V. Currents were held at +60 mV and stepwise 500ms pressure pulses from 0 to -90mmHg in -10mmHg increments were made. The red trace depicts the current elicited at -50 mmHg.

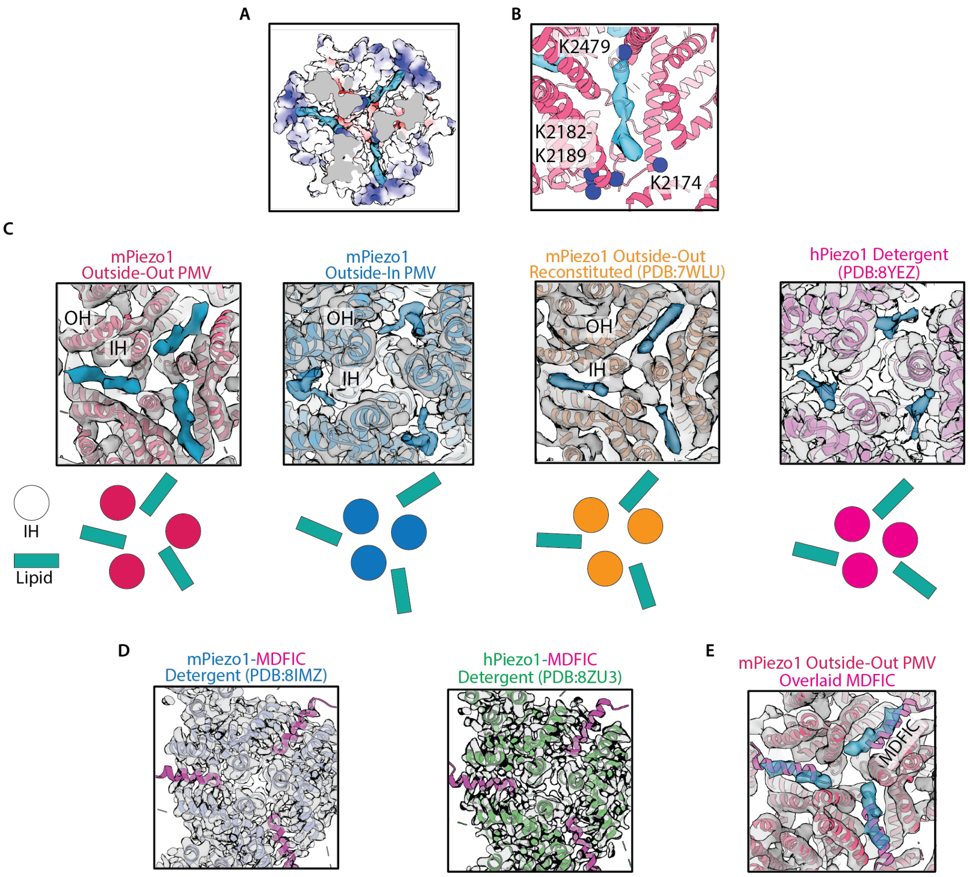

Fig. S10. Focus on the Piezo1 regulatory binding site. (A) Top-own view of the outside-out PMV Piezo1 transmembrane pore from at the regulatory binding site, depicting the electrostatic surface, where blue is electropositive, white is neutral and red is electronegative. The cryo-EM map of the putative cofactor is included. (B) A close-up view of the binding site, depicting the positions of proximal lysine residues as blue spheres relative to the cryo-EM map of the putative cofactor. (C) Top-down views of the Piezo1 transmembrane pore, highlighting the location of additional densities that have been described as "pore lipids" relative to the IH and OH pore helices in ours and other group's cryo-EM structures of Pizo1. Below each annotated structure is a schematic illustrating the relative position of the putative lipid to the IH. (D) A top down view of the binding site of MDFIC to Piezo1 in mouse (left) and human (right) Piezo1-MDFIC complex structures. (E) A top-down view of the regulatory binding site in the outside-out PMV Piezo1 structure and the putative lipid density (blu) with the C-terminal helix of MDFIC from a mouse Piezo1-MDFIC complex structure (PDB:8IMZ) aligned and shown in pink.

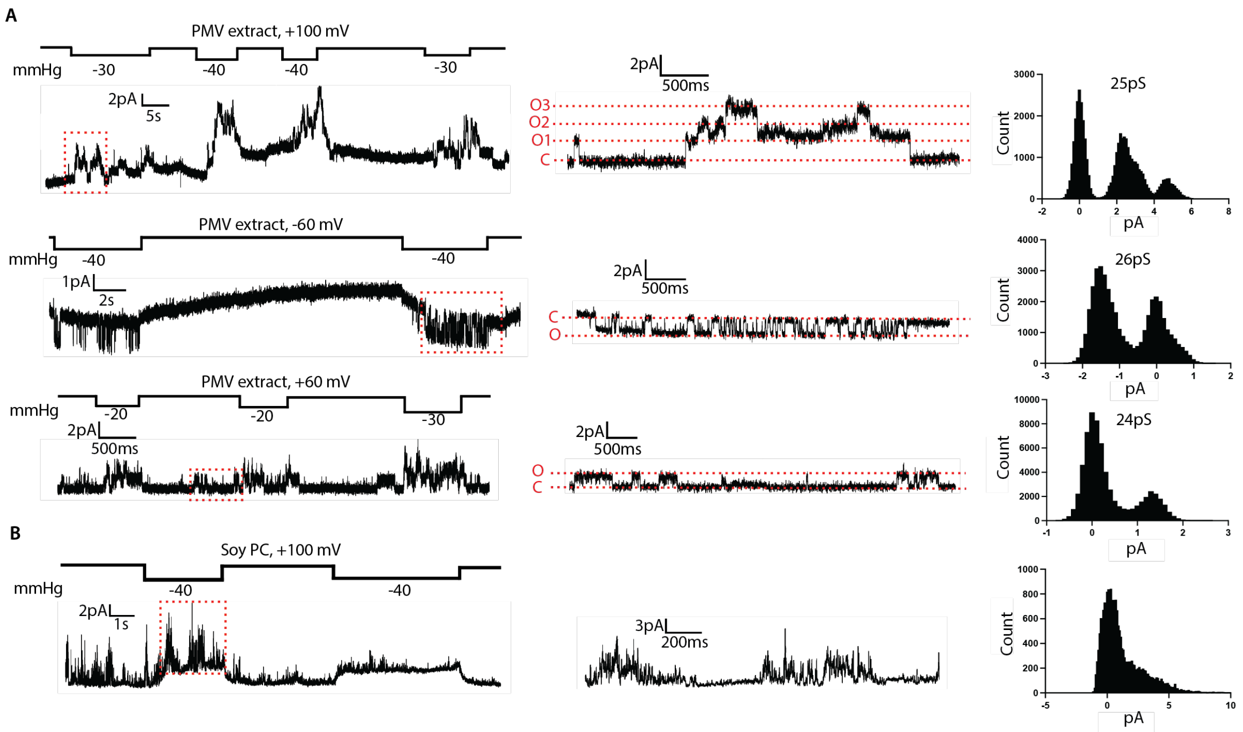

Fig. S11. Electrical recordings of purified Piezo1 reconstituted into GUVs. (A) Representative electrical recordings of patches excised from PMV extract GUVs fused with Piezo1 liposomes. Gap-free recordings are shown (left) and a close-up of single channel openings are adjacent, which are derived from the red-dashed box. (right) Amplitude histograms are shown and the measured single channel conductance value. (B) As in *A* but for recordings made from soy PC GUVs after fusion with Piezo1 liposomes.

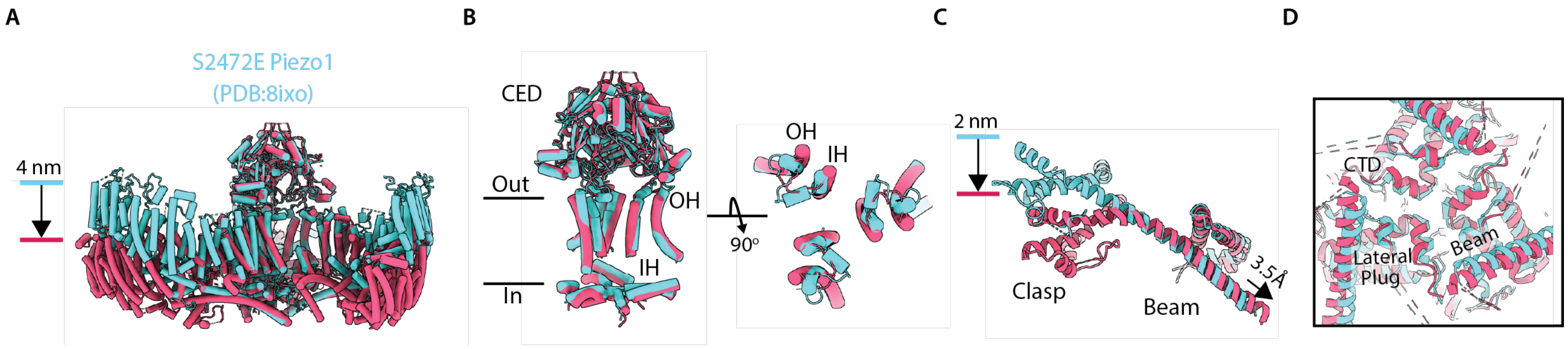

Fig. S12. Comparison of outside-out PMV Piezo1 and a Piezo1 mutant, S2472E (A) Aligned structures of outside-out PMV (red) and Piezo1 mutant S2472E (cyan). The major displacement of the transmembrane arms is highlighted. (B) Regions of Piezo1 involved in pore-gating are aligned from a side-view (left) and top-down view with a close-up of the pore-lining helices (right). (C) An isolated view of the Piezo1 beam helix, clasp and CTD region of the two structures. (D) A close-up view of the beam, CTD and lateral plug that has been proposed to form the cytosolic gate for ion conduction in Piezo1, is shown. The sliding of the beam helix is associated with conformational changes at this region in the outside-out Piezo1 structure and not the mutant structure.

Piezo1 Expression	Piezo1 purification	Lipids for reconstitution	Experimental Type	Success	Comment
mPiezo1-GFP in HEK293 GnTI- cells	Anti-GFP resin. C12E10 for solubilization and purification	Soy PC	GUVs formed by dehydration and rehydration method	No	
mPiezo1-GFP in HEK293 GnTI- cells	Anti-GFP resin. C12E10 for solubilization and purification	3:1:1 POPE:POPC:POPS	GUVs formed by dehydration and rehydration method	No	
mPiezo1-GFP in HEK293 GnTI- cells	Anti-GFP resin. C12E10 for solubilization and purification	8:1:1 POPC:DOPS:Cholesterol	GUVs formed by dehydration and rehydration method	No	Rare spontaneous openings but short-lived
mPiezo1-GFP in HEK293 GnTI- cells	Anti-GFP resin. CHAPS for solubilization and purification	Soy PC	GUVs formed by dehydration and rehydration method	No	
mPiezo1-GFP in HEK293 GnTI- cells	Anti-GFP resin. C12E10 for solubilization and purification	Brain extract polar	GUVs formed by dehydration and rehydration method	No	Very few GUVs, hard to form gigaseal
mPiezo1-GFP in HEK293 GnTI- cells	Anti-GFP resin. C12E10 for solubilization and purification	Soy PC	GUVs formed by gel-assisted swelling	No	Occasional observation of very large porin-looking currents
mPiezo1-GFP in HEK293 GnTI- cells	Anti-GFP resin. C12E10 for solubilization and purification	Soy PC	Horizontal bilayer composed of 3:1:! POPE:POPC:POPS	No	
mPiezo1-ALFA-GFP in HEK293 GnTI+ cells	Anti-ALFA resin, C12E10 for solubilization and purification	Soy PC	GUVs formed by dehydration and rehydration method	No	Leaky currents, not reversibly activated by pressure at high prot:lipid ratios
mPiezo1-ALFA-GFP in HEK293 GnTI+ cells	Anti-ALFA resin, C12E10 for solubilization and purification	Soy PC	GUVs formed by PEG-mediated fusion of Piezo1 liposomes and soy PC GUVs	No	Only noisy non-channel currents at high prot:lipid ratio
mPiezo1-ALFA-GFP in HEK293 GnTI+ cells	Anti-ALFA resin, C12E10 for solubilization and purification	Soy PC	GUVs formed by PEG-mediated fusion of Piezo1 liposomes and PMV extract GUVs	Yes	Still limited by successful number of patches

Table S1. Summary of experimental conditions used to assess reconstituted Piezo1 function.

**Collection Parameters**	*mPiezo1*<br>in plasma membrane vesicles<br>Outside-In Orientation<br>PDB ID 11YE<br>EMD-76184	*mPiezo1*<br>in plasma membrane vesicles<br>Ouside-Out Orientation<br>PDB ID 11ZC<br>EMD-76212
Accelerating Voltage (kV)	300	300
Number of frames	1,953	1,953
Dose (e^{-}/Å^2)	60	60
Defocus Range (µm)	-1.5 to -2.5	-1.5 to -2.5
Exposure Time (s)	6.119	6.119
Original Pixel size (Å)	0.94	0.94
Spherical aberration (mm)	2.7	2.7
Amplitude Contrast	0.07	0.07
**Map Parameters**		
Final Pixel Size (Å)	0.94	1.88
Symmetry	C3	C3
Total Micrographs	18,480	16,060
Initial Particles	529,310	909,080
Final Particles	69,370	11,478
Map Resolution (Å)	3.7	6.6
FSC threshold	0.143	0.143
**Model Composition**		
Nonhydrogen atoms	29,511	16,692
Protein residues	4,056	4,173
Ligands		0
r.m.s.d. bond length (Å)	0.002	0.004
r.m.s.d. bond angle	0.552	0.984
**Validation**		
MolProbity Score	2.08	1.75
Clash Score	11.58	2.67
Poor Rotamers (%)	0.64	0.00
Ramachandran Plot		
Favored (%)	91.49	82.93
Allowed (%)	8.21	15.57
Disallowed (%)	0.3	1.5

Table S2. Cryo-EM data collection, map parameters and validation statistics

Movie S1.

Movie showing morph transitions between curved and flat Piezo1 structures from cell-derived membranes. Regions important for channel gating are color-coded and annotated.

.
